# Unveiling an Uncommon Glucosylceramidase (GBA) Mutation: Gaucher Disease Due to p.Ser276Phe Substitution

**DOI:** 10.7759/cureus.101435

**Published:** 2026-01-13

**Authors:** Naveen Kumar, Prasad Dange, Amrapali Samadder, Shabnam Kalita, Jaya Shankar Kaushik

**Affiliations:** 1 Pathology and Laboratory Medicine, All India Institute of Medical Sciences, Guwahati, Guwahati, IND; 2 Pathology, All India Institute of Medical Sciences, Guwahati, Guwahati, IND; 3 Pediatrics, All India Institute of Medical Sciences, Guwahati, Guwahati, IND

**Keywords:** bone marrow aspiration and biopsy, gaucher disease type 3, gba gene mutation, lysosomal storage diseases, splenomegaly

## Abstract

Gaucher disease (GD) is an autosomal recessive lysosomal storage disorder resulting from pathogenic variants in the *GBA1* gene, which encodes the enzyme glucocerebrosidase. We describe a child with neuropathic GD (type 3) associated with an uncommon *GBA1* variant, p.Ser276Phe. A four-year-old girl, born to non-consanguineous parents, presented with gradually progressive neurological symptoms accompanied by systemic involvement. Examination revealed marked splenomegaly. Bone marrow biopsy demonstrated extensive infiltration by macrophages with characteristic wrinkled, fibrillary cytoplasm, partially replacing the marrow spaces, raising suspicion of GD. Antiepileptic therapy with levetiracetam resulted in partial improvement of neurological manifestations. Whole-exome sequencing identified a homozygous missense variant in exon 7 of the *GBA1* gene, leading to the substitution of phenylalanine for serine at codon 276, within the PF07714 protein kinase domain. On follow-up at six months, the child continued to exhibit myoclonic jerks, progressive ataxia, and cognitive decline, consistent with a neuropathic disease course.

While p.Leu483Pro is the most frequently reported mutation in the Indian population and is often associated with severe neurological disease in homozygous individuals, the p.Ser276Phe variant has been documented only rarely in the literature. This case highlights an uncommon *GBA1* mutation and further illustrates the wide phenotypic variability and unpredictable clinical expression seen in neuropathic GD.

## Introduction

Gaucher disease (GD), an autosomal recessive disorder, results from a mutation in the glucosylceramidase beta gene (GBA1; OMIM *606463), which encodes the enzyme glucocerebrosidase (EC 3.2.1.45; also known as acid β-glucosidase and GCase). It has a prevalence of 1:500000 in the general population and 1:950 live births in the Ashkenazi Jewish population [1,2].

The human GBA1 gene, which is found on chromosome 1q21 and spans 7.6 kb of genomic DNA, has 12 exons and 11 introns. There is a highly homologous pseudogene (GBAP) 16 kb downstream [3]. Recombination events between GBA1 and GBAP are facilitated by their close proximity and high degree of sequence identity [4]. GD shows considerable clinical variability and is traditionally divided into three types according to the extent of neurological involvement. Type 1 is the non-neuronopathic form; neurological features are absent, and affected individuals may remain asymptomatic or present at any stage of life. In contrast, both type 2 and type 3 diseases are associated with neurological manifestations beginning in infancy, although only type 2 follows a rapidly progressive course leading to early mortality [5]. Here, we describe a four-year-old child with Gaucher disease type 3 (GD3) who presents with a rare p.Ser276Phe mutation.

## Case presentation

A previously healthy four-year-old girl, born to non-consanguineous parents, was brought in with gradually progressive neurological and systemic complaints. Symptoms first became apparent at around two years of age, when she developed early-onset ataxia and oculomotor apraxia. By the age of three years, she began experiencing myoclonic jerks, followed by a steady decline in motor abilities and cognition. On examination, she was found to have marked splenomegaly measuring approximately 11 cm. Neuroimaging and fundoscopic evaluation were unremarkable. 

**Figure 1:**
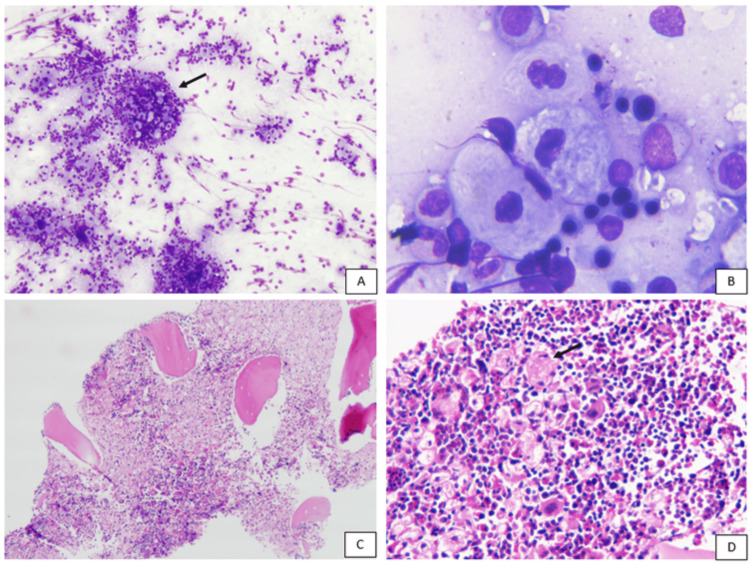
Bone marrow aspirate smear and biopsy A: Bone marrow aspirate smear with Gaucher cells (in cluster, arrow) (x100, MGG stain). B: Gaucher cell with a typical wrinkled paper appearance (x1000, oil immersion). C and D: Bone marrow biopsy presenting with several Gaucher cells (arrow) along with other hematopoietic precursors respectively (x40 and x400, Hematoxylin and Eosin stain).

Hematological evaluation showed anemia and leukopenia with preserved platelet counts and no significant morphological abnormalities. A bone marrow aspirate and biopsy were planned and performed, which demonstrated extensive infiltration by sheets of macrophages with characteristic wrinkled, tissue-paper-like cytoplasm and fibrillary appearance, partially replacing the marrow spaces, with residual foci of hematopoiesis (Figure 1).

A diagnosis of GD was suggested. The child was started on levetiracetam, resulting in partial control of myoclonic jerks; however, she subsequently had three episodes of generalized tonic-clonic seizures and was referred for hormonal therapy. Whole-exome sequencing identified a homozygous missense variant in exon 7 of the GBA1 gene (chr1:g.155237513G>A; Depth: 118x), resulting in the substitution of phenylalanine for serine at codon 276 (p.Ser276Phe; ENST00000368373.8) (Table 1).

**Table 1:** Whole-exome sequencing Whole-exome sequencing report of the patient, showing GBA1 mutation (p.Ser276Phe substitution) along with SPTBN2 mutation (p.Val994Leu) associated with type 3 GD. GD: Gaucher disease

Gene (Transcript)	Location	Variant	Zygosity	Disease (OMIM)	Inheritance	Classification
GBA1 - (ENST00000368373.8)	Exon 7	c.827C>T (p.Ser276Phe)	Homozygous	Gaucher disease, type I, II, III, IIIC (OMIM#230900) /(OMIM#231000) /(OMIM#231005)	Autosomal recessive	Likely pathogenic (PS4,PM2,PP2)
SPTBN2 (-) (ENST00000533211.6)	Exon 17	c.2980G>T (p.Val994Leu)	Heterozygous	Spinocerebellar ataxia 5 (OMIM#600224) / Spinocerebellar ataxia- 14 (OMIM#615386)	Autosomal dominant	Uncertain significance (PM2)

This variant is located within the "Protein kinase domain|PF07714" of the GBA1 protein. At the recent six-month follow-up, the patient continued to exhibit persistent myoclonic jerks, progressive ataxia, and further cognitive decline, in keeping with a neuropathic form of GD.

## Discussion

GD represents one of the most extensively studied lysosomal storage disorders; however, it continues to pose diagnostic and prognostic challenges because of marked molecular heterogeneity and variable clinical expressivity. More than 500 pathogenic variants have been described in the GBA1 gene. p. Leu483Pro is the first mutation reported in GD [6]. Despite this extensive cataloguing, clear genotype-phenotype correlations remain elusive, particularly for the neuropathic forms of GD [7].

The present case describes a child with a progressive neuronopathic phenotype consistent with GD type 3, harbouring a rare homozygous missense mutation, p.Ser276Phe, in exon 7 of GBA1. This variant has been reported only sparsely in the literature and remains poorly characterized in terms of clinical correlates [8]. The early onset of neurological manifestations in our patient, including oculomotor apraxia, progressive ataxia, myoclonic epilepsy, and cognitive decline, together with significant visceral involvement, underscores the severe disease course associated with this mutation.

In the Indian population, p.Leu483Pro is the most frequently encountered pathogenic variant and is often associated with severe phenotypes, including neurological involvement, particularly in the homozygous state, and the carrier frequency of p.Leu483Pro was reported to be 1:600, thus making it the commonest mutation [9-11]. Other mutations, such as p.Val433Leu, p.Gly416Ser, and p.Asn227Ser, have also been implicated in neuronopathic GD [12]. In contrast, p.Ser276Phe has been reported only in a single large Indian cohort study, without detailed phenotypic correlation. Its absence in several large mutational spectrum studies across diverse populations, including Thai, Brazilian, Romanian, Japanese, Chinese, Ashkenazi Jewish, and European cohorts, highlights its rarity and potential population-specific occurrence [13].

The p.Ser276Phe variant results in the substitution of a polar serine residue with a bulky, hydrophobic phenylalanine within the PF07714 protein kinase-like domain of glucocerebrosidase. Although glucocerebrosidase is not a kinase, this conserved domain is believed to play a role in protein folding, stability, or intracellular trafficking. Substitution at this site may therefore adversely affect enzyme conformation or lysosomal targeting, leading to reduced residual enzymatic activity and accumulation of glucosylceramide in macrophages and neuronal tissue. This molecular disruption plausibly explains the combined visceral and neurological phenotype observed in our patient.

Notably, despite symptomatic antiepileptic therapy, the child demonstrated continued neurological deterioration over a short follow-up period, reflecting the progressive nature of neuronopathic GD and the limited efficacy of supportive therapy in halting neurodegeneration. This observation further emphasises the importance of early molecular diagnosis, genetic counselling, and timely consideration of disease-modifying therapies where available.

From a diagnostic standpoint, this case reiterates the value of integrating classical histopathological findings, such as Gaucher cells in bone marrow, with advanced molecular testing. In regions with diverse or under-reported mutational spectra, reliance on targeted mutation panels alone may result in missed or delayed diagnoses. Whole-exome sequencing or comprehensive GBA1 gene analysis is therefore particularly valuable in pediatric patients with atypical or progressive neurological features.

Few large studies are summarized in Table 2 with the GBA gene mutational spectrum.

**Table 2:** Studies on the GBA gene mutational spectrum

Studies on Gaucher's disease	Number of patients	GBA mutational spectrum
Phetthong et al. [14]	27	14 variations were found, including 12 known pathogenic variants (p.L483P, p.N409S, p.R159W, p.P305A, p.A175G, p.D448H, p.V414L, IVS2+1G>A, IVS6–1G>C, IVS7+1G>C, IVS9–3C>G, and Rec1a). The most common allele identified in this investigation was p.L483P; however, no occurrences of p.Ser276Phe were detected.
Rozenberg et al. [8]	247	Mutation p.Gly416Ser is observed at high frequency in Brazilian GD type 3 GD patients, but p.Ser276Phe was not reported in any of the cases.
Choy et al. [15]	27	Examined 29 Gaucher disease cases. The mutations R353W (c.1174C>T), F37V/L444P, G46E/L444P, R48W/R120W, N188S/L444P, Y205C/L444P, N370S/L444P, F213I, L385P, P122L, V375L, Y363C, M416V, and 383 400del were present, but p.Ser276Phe was not reported in any of these mutations.
Bisariya et al. [16]	24	About half of the patients had mutant alleles p.Leu483Pro, p.Asn409Ser, IVS2 +1G>A, p.Asp448His, and c.1263_1317del (55Del); p.Leu483Pro was the most prevalent mutation; however, p.Ser276Phe was not found in any of the cases.
Sheth et al. [17]	100	Found 33 mutations in 100 individuals, including four missense variants (p.Ser136Leu, p.Leu279Val, p.Gly383Asp, p.Gly399Arg). The most prevalent mutation found in the study (62% of patients) was p.Leu483Pro. Two patients were documented to have p.Ser276Phe. This study did not describe any particular clinical characteristics linked to this mutation.
Wan et al. [18]	208	p.Ser276Phe was not reported in any of the instances; however, p.Phe252Ile and p.Leu483Pro (RecNciI) were found in Chinese and Japanese patients.
Giraldo et al. [19]	193	Ashkenazi Jewish, Portuguese, and Spanish patients had p.Asn409Ser, but none of the cases had p.Ser276Phe.
Stone et al. [4]	31	Point mutations, splice junction mutations, deletions, fusion alleles, and recombinant alleles were among the thirty-three distinct mutant alleles discovered. These patients have 11 new mutations: R131L, H255Q, R285H, S196P, H311R, c.330delA, V398F, F259L, c.533delC, Y304C, and A190E. On 25 patient alleles, the mutation L444P was discovered. Mutation L444P occurred alone on nine alleles, with E326K on one allele and as part of a recombinant allele on fifteen alleles, according to Southern blots and direct sequencing. However, p.Ser276Phe was not reported in any of the cases.
Dimitriou et al. [6]	141	Greek ancestry accounted for 111/141 (78%) of the GD patients. Albanian (24/141; 17%), Syrian (2/141; 1.4%), Egyptian (2/141; 1.4%), Italian (1/141; 0.7%), and Polish (1/141; 0.7%) were the remaining patients. 37 distinct genotypes and 28 distinct mutations were found by mutation analysis. Seven of the alterations (T231I, D283N, N462Y, LI75P, F81L, Y135S, and T482K) had never been reported before. N370S, D409H, H255Q, and L444P were the most common mutations. Only Greek and Albanian patients had the mutation D409H;H255Q; p.Ser276Phe was not found in any of the cases.
Kawame et al. [20]	8	Discovered that all three clinical subgroups of Japanese Gaucher disease patients had the 1448C and 754A mutations. Only two nonneuronopathic patients (ages 1 year 6 months and 7 years) had homozygosity for the 1448C mutation, which was detected on 12 (40%) of the 30 chromosomes (44% allele frequency in nonneuronopathic form and 33% in neuronopathic forms). Six (20%) of the thirty chromosomes had the 754A mutation. The 754A mutation was not homozygous in any patient. Additionally, they found four patients who were compound heterozygotes for 754A and 1448C, although none of the cases had p.Ser276Phe.
Koprivica et al. [13]	128	Over 97% of the mutant alleles were found. p.Ser276Phe was not found in any of the 14 new mutations (A90T, N117D, T134I, Y135X, R170C, W184R, A190T, Y304X, A341T, D399Y, c.153-154insTACAGC, c.203-204insC, c.222-224delTAC, and c.1122-1123insTG.

## Conclusions

This case highlights a rare and poorly characterized GBA1 mutation, p.Ser276Phe, associated with severe neuronopathic GD presenting in early childhood. The findings expand the existing mutational and clinical spectrum of GD and underscore the unpredictable genotype-phenotype relationships inherent to GBA1 mutations. Recognition of such uncommon variants is crucial for accurate diagnosis, prognostication, and genetic counseling, particularly in populations with heterogeneous genetic backgrounds.
